# DNA Repair Gene XRCC4 Codon 247 Polymorphism Modified Diffusely Infiltrating Astrocytoma Risk and Prognosis

**DOI:** 10.3390/ijms15010250

**Published:** 2013-12-27

**Authors:** Zhong-Hui Lin, Jin-Chun Chen, Yun-Sun Wang, Teng-Jiao Huang, Jin Wang, Xi-Dai Long

**Affiliations:** 1 Department of Neurology Medicine, Xia-Men Hospital of Traditional Chinese Medicine, Xiamen 361009, China; E-Mail: forestlzh@aliyun.com; 2 Department of Medicine, Xia-Men Hospital of Traditional Chinese Medicine, Xiamen 361009, China; E-Mails: fjchenjc@163.com (J.-C.C.); fjxmwangsy@163.com (Y.-S.W.); xmwangtj@163.com (T.-J.H.); 3 Department of Neurology Medicine, Guangxi Medical University, Nanning 530021, China; E-Mail: gxwjmc1986@163.com; 4 Department of Pathology, Youjiang Medical College for Nationalities, Baise 533000, China; 5 Department of Liver Surgery, Ren Ji Hospital, School of Medicine, Shanghai Jiao Tong University, Shanghai 200127, China

**Keywords:** XRCC4, polymorphism, DIA

## Abstract

The DNA repair gene X-ray cross-complementary group 4 (XRCC4), an important caretaker of the overall genome stability, is thought to play a major role in human tumorigenesis. We investigated the association between an important polymorphic variant of this gene at codon 247 (rs373409) and diffusely infiltrating astrocytoma (DIA) risk and prognosis. This hospital-based case-control study investigated this association in the Guangxi population. In total, 242 cases with DIA and 358 age-, sex-, and race-matched healthy controls were genotyped using TaqMan-PCR technique. We found a significant difference in the frequency of XRCC4 genotypes between cases and controls. Compared with the homozygote of XRCC4 codon 247 Ala alleles (XRCC4-AA), the genotypes of XRCC4 codon 247 Ser alleles (namely XRCC4-AS or -SS) increased DIA risk (odds ratios [OR], 1.82 and 2.89, respectively). Furthermore, XRCC4 polymorphism was correlated with tumor dedifferentiation of DIA (*r* = 0.261, *p* < 0.01). Additionally, this polymorphism modified the overall survival of DIA patients (the median survival times were 26, 14, and 8 months for patients with XRCC4-AA, -AS, and -SS, respectively). Like tumor grade, XRCC4 codon 247 polymorphism was an independent prognostic factor influencing the survival of DIA. These results suggest that XRCC4 codon 247 polymorphism may be associated with DIA risk and prognosis among the Guangxi population.

## 1. Introduction

Tumors from the central nervous system represent approximately 2% of all cancers, with an estimated 4.2 to 5.4 per 100,000 individuals affected per year [1,2]. Among these tumors, diffusely infiltrating astrocytoma (DIA) is the most common type, which comprises approximately 60% of primary brain tumors [1,3]. In relatively high incidence areas of DIA, such as Guangxi Zhuang Autonomous region, China, this tumor accounts for 70 percent of primary brain tumors. According to our not published epidemiological data, during May 2005 to April 2008, incidence rates and death rates of DIA were about 10/10,000 and eight to nine per 10,000 in some areas of Guangxi, respectively.

DIA varies considerably in its epidemiological features, morphologic attributes, growth patterns, genetic profiles, and clinic behavior. Histopathologically, this tumor is divided into three groups: low-grade astrocytoma (the World Health Organization [WHO] Grade II), anaplastic astrocytoma (WHO Grade III), and glioblastoma (WHO Grade IV) [3]. Despite improvements in diagnosis and treatment, the prognosis of DIA remains dismal with a 5-year survival rate of less than 30% [1]. There is a paucity of information on the etiology of this tumor. Epidemiological studies have shown that occupations, environmental carcinogens, diet, and ionizing radiation can elevate DIA risk [1–5]. These factors can induce various types of DNA damage, including DNA double-strand breaks (DSBs) [6–8]. This suggests that an individual susceptibility related to genetic factors (e.g., DNA repair capacity) might be associated with DIA tumorigenesis [3]. The nonhomologous end-joining repair (NHEJ) genes play an important role in the repair of DSBs resulting from exogenous attacks [9,10], and might be important cancer-correlated genetic factors [6,11–13].

While DNA repair gene x-ray repair cross-complementary Group 4, a key component of NHEJ pathway, is found to restore DNA double-strand break repair and has the ability to support V(D)J recombination [14–16]. The genetic polymorphism at the code 247 of XRCC4 gene (rs3734091) has been reported to be associated with DNA repair capacity and various cancers [17], indicating that it might play an important role in various cancers [15,17–20]. However, the association between it and DIA has not yet been elucidated. Therefore, we specifically conducted a hospital-based case-control study to examine whether this polymorphism modified the risk and the prognosis of DIA among Guangxi population.

## 2. Results

### 2.1. Demographic and Clinic Characteristics of the Subjects

Our final analysis included 242 DIA cases and 358 controls. The demographic characteristics of all cases and controls are shown in Table 1. The mean age, gender ratio, and race distribution are of the same levels in both control and DIA groups (*p* > 0.05). According to the WHO grading standard of DIA, cases with IV-grade tumors accounted for 58.7% (142/242), whereas the frequencies of II-grade, and III-grade cases were 19.4% (47/242) and 21.9% (53/242), respectively.

### 2.2. XRCC4 Codon 247 Polymorphism Increased DIA Risk

The genotypic distribution of XRCC4 codon 247 for both tumor patients and controls is summarized in Table 2. The frequencies of the codon 247 Ser allele among cases and controls were 22.3% and 11.3%, respectively. Genotype frequent distribution in controls fit the Hardy-Weinberg equilibrium well. The heterozygous genotype with XRCC4 codon 247 Ala and Ser allele (XRCC4-AS) and the variant homozygous genotype with XRCC4 codon 247 Ser allele (XRCC4-SS) were more frequent among cases than the controls (*p* < 0.01), resulting in an Ser allele frequency of 22.3% in cases and 11.3% in controls. Logistic regression analysis showed that the adjusted odds ratio (OR) for DIA for those individuals carrying XRCC4-AS compared with those exhibiting the homozygote for Ala alleles (XRCC4-AA) was 1.82 (95% confidence interval [CI], 1.13–2.94), and the corresponding OR for those featuring XRCC4-SS was 2.89 (95%CI, 1.62–15.15). These results showed that DIA risk was associated with the number of codon 247 Ser alleles.

To assess possible interactive effects of matching factors and XRCC4 codon 247 polymorphism on DIA risk, we performed a series of bivariate stratified analyses by matching factors (including age, race, and sex), and did not find that these factors modulated the effect of this polymorphism on DIA risk (*P*_interaction_ > 0.05; Table 3). This implied that these matching factors should be effectually manipulated and should not modify the association between this polymorphism and DIA risk.

### 2.3. XRCC4 Codon 247 Polymorphism Correlated with the Tumor Grade of DIA

To investigate whether XRCC4 codon 247 polymorphism correlated with the clinic pathological features of DIA, an association analysis of the risk genotypes [*i.e.*, genotypes of the XRCC4 codon 247 Ser alleles (XRCC4-AS/SS)] or the non-risk genotype (XRCC4-AA) and the clinical pathological characteristics of DIA was first performed separately. Significant differences in the distributions of the genotypes were observed with respect to different tumor grades but not with respect to age, gender, and minority status (data not shown). Spearman *r* test next exhibited that XRCC4 codon 247 polymorphism was positively related to a higher grade of DIA (*r* = 0.261, *p* < 0.01; Table 4). Taken together, this polymorphism might correlate with the progression of DIA from low grade to high grade.

### 2.4. XRCC4 Codon 247 Polymorphism Modified DIA Prognosis

**Figure 1.**
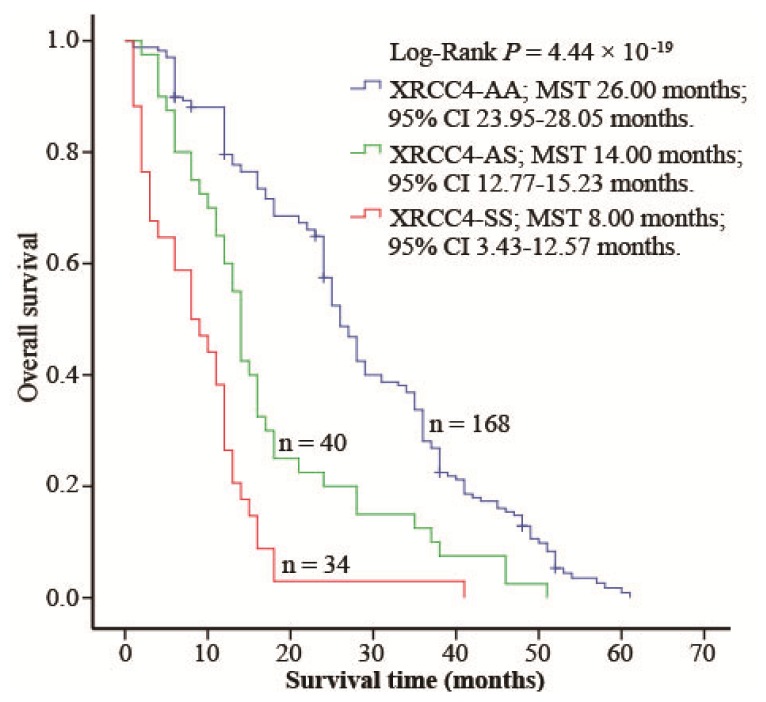
XRCC4 codon 247 polymorphism was correlated with the overall survival (OS) of diffusely infiltrating astrocytoma (DIA). Cumulative hazard function was plotted by the Kaplan-Meier methodology and the *P* value was calculated with two-sided log-rank tests. MST, the median survival time.

Because a previous study had shown that the variants in the DNA repair genes might modify DIA prognosis [21], we analyzed the survival information of all DIA cases. During the follow-up period of 242 DIA patients, 231 died 77.2% of which showed a one-year overall survival rate (OSR), 25.8% a three-year OSR, and 0.6% a five-year OSR. Kaplan–Meier survival analysis showed that DIA patients carrying XRCC4-AS/SS had a shorter overall survival time [the median survival time (MST) was 14 and 8 months for patients with XRCC4-AS and XRCC4-SS, respectively] compared to those with XRCC4-AA (MST, 26 months) (Figure 1). Multivariate cox regression analysis (with stepwise forward selection based on likelihood ratio test) was next performed to determine whether XRCC4 codon 247 polymorphism was an independent predictor of overall survival for patients with DIA. The results exhibited that this polymorphism increased the risk of dying of DIA, with a hazard ratio (HR) of 2.26 for XRCC4-AS and 5.36 for XRCC4-SS (*p* < 0.01; Table 5), respectively.

## 3. Discussion

To the best of our knowledge, no studies have investigated the role of DNA-repair gene XRCC4 for patients suffering from DIA, especially in the epidemic areas. In this study, we analyzed the association between XRCC4 codon 247 Ala > Ser polymorphism and the risk of DIA among the Guangxi population in an area with a relatively high incidence of DIA. The results showed that XRCC4 codon 247 Ser alleles had a substantial association with the increasing risk of DIA (adjusted OR 1.82 for XRCC4-AS; 2.89 for XRCC4-SS). These results suggest that XRCC4 codon 247 polymorphism may have functional significance in DIA.

XRCC4, located on chromosome 5q14.2, is an important DNA repair gene involved in the NHEJ pathway [15,22]. The encoded protein of XRCC4 interacts directly with Ku70/Ku80 and plays a central role in the precise end-joining of blunt DSBs [10,22]. Mutations in the coding region of this gene might result in more deficient NHEJ capacity [23–25] and increase cancer risk [17–20]. To date, more than forty genetic polymorphisms based on mutations have been found in XRCC4, some of which are related to malignant tumors such as hepatocellular carcinoma [18,19], gastric cancer [26,27], oral cancer [20], bladder cancer [28], and esophageal cancer [29]. In this study, we only analyzed XRCC4 codon 247 polymorphism, mainly because this polymorphism localizes at conserved sequence of XRCC4 and results in the missense mutation Ala to Ser. This suggests that the missense mutation at codon 247 of XRCC4 codon 247 might decrease DNA repair capacity and increase tumor risk. Our present study exhibited that this polymorphism elevated DIA risk in the Guangxi population. Similar to our results, Tseng *et al.* [20] investigated the effects of XRCC4 codon 247 polymorphism on oral cancer in Taiwan (another Chinese population) and found that persons carrying XRCC4-AS had a higher cancer risk than those with XRCC4-AA (adjusted OR = 2.04, *p* = 0.023). Recently, a large hospital-based case-control epidemiologic study has also shown that this polymorphism increases the risk of hepatocellular carcinoma [18]. Furthermore, some interactive evidence of mutative alleles and genetic toxin is observed in this epidemiological study.

Taken together, those results suggest that XRCC4 codon 247 polymorphism may alter the normal protein function and, consequently, may be associated with a reduction in DNA repair capacity [17]. Thus, the DNA damage from environmental risk factors cannot be repaired effectively and duly, consequently it may induce genetic mutation [6], and brain cell canceration. Thus, this polymorphism may play a role in the carcinogenetic pathway of Guangxiese DIA.

Because previous studies had shown that XRCC4 codon 247 polymorphism may modify tumor outcome [18,21], we analyzed the relationship between this polymorphism and DIA prognosis. We found that XRCC4 codon 247 polymorphism shortened MST of DIA (14 months for XRCC4-AS and 8 months for XRCC4-SS) and increased death risk of DIA (HRs were 2.26 and 5.36 for XRCC4-AS and -SS, respectively), possibly because it correlates with the fact that this polymorphism modifies tumor grade and differentiation, and, consequently, might promote tumor metastasis. Supporting our results, recent studies have exhibited that XRCC4 codon 247 polymorphism is associated with a decreased XRCC4 expression [18], and that down-regulated expression of XRCC4 modifies DIA prognosis [21].

In the present study, to control the effects of confounders such as age, gender, and race, we used an individually matched design. In the stratified analysis, no interactive effects were found, suggesting that these factors do not modify the correlation between XRCC4 codon 247 polymorphism and DIA.

However, there were several limitations to our study. Potential selection bias might have occurred because the selection of control subjects in our study was hospital-based. Despite the analysis of the XRCC4 codon 247 polymorphism, we did not analyze other polymorphisms of this gene possibly able to modify the risk of DIA. Our findings were based on relatively small numbers and limited by small number subjects in part of the genotype strata. Therefore, more genes deserve further elucidation based on a large sample and the combination of genes.

## 4. Experimental Section

### 4.1. Study Population

Between January 2004 and December 2009, incident DIA cases were recruited at the affiliated hospitals of two main medical universities in Guangxi, Guangxi Medical University and Youjiang Medical College for Nationalities. All cases were histopathologically confirmed and previously treated with chemotherapy or radiotherapy. There were no age, sex, race, or tumor stage restrictions. Each new patient was screened with a brief eligibility questionnaire that assessed prior tumor therapy and willingness to participate in this study by the instituted staff interviewers. If the patient was willing to participate, the interviewer accompanied the study participant to a private room to conduct the interview. Healthy control subjects without a history of cancer were randomly selected from a pool of healthy volunteers. Because of the routine scheduled physical exams, volunteers visited the general health check-up centers of the same hospitals. The controls were individually matched (1:1 or 2:1) to cases based on ethnicity (Han, Minority), sex, and age (±5 years). Every potential control was first surveyed by using a short questionnaire to elicit willingness to participate in the study and to provide preliminary demographic data for matching. A total of 242 cases and 358 controls, representing 95% of eligible cases and 98% of eligible controls, were enrolled and interviewed. After giving written consent, demographic information and clinical pathological data were collected in the hospitals using a standard interviewer administered questionnaire and/or medical records. At the same time, 4 mL of peripheral blood was obtained for DNA extraction. This study was approved by the ethics committees of the hospitals involved in this study. All activities involving human subjects were carried out under full compliance with government policies and the Helsinki Declaration.

### 4.2. DNA Extraction

DNA was extracted from peripheral blood samples from all tumor patients and control subjects in a 1.5 mL microcentrifuge tube for deparaffinization and proteinase K digestion, as described by standard procedures (Protocol #BS474, Bio Basic, Inc., Markham, ON, Canada). DNA was stored at 4 °C until additional analysis.

### 4.3. Genotyping

Genotypes were analyzed by using a previously published TaqMan-PCR technique [18,19]. Briefly, PCR analysis was accomplished on iCycle iQ™ real-time PCR detection system (iQ5, Bio-Rad, Hercules, CA, USA). The following primers and primers were used: 5′-TGAGGAAAGTGAAAACCAA ACTGATCT-3′, 5′-GCCCAAATAAGATATTCAACAGAGGAGAT-3′, 5′-FAM-CCTGAAGACAAC CC-MGB-3′, and 5′-HEX-CCTGAAGCCAACCC-MGB-3′ [18]. All primers and probes were synthesized by Shanghai GeneCore BioTechnologies Co., Ltd. (Shanghai, China). Each PCR was carried out in total volume of 5 μL consisting of 1× TaqMAN Universal Master Mix II (cat#4440041, Applied Biosystems, Carlsbad, CA, USA), 0.2 μM of each probe, 0.2 μM of each primer, and about 15 ng of genomic DNA. Cycling conditions were 95 °C for 30 s, and 50 cycles of 95 °C for 15 s and 60 °C for 1 min. For quality control, laboratory personnel were blinded to case and control status. Controls were included in each run, and repeated genotyping and sequencing of a random 20% subset yielded 100% identical genotypes.

### 4.4. DIA Patients Follow-up

All DIA patients were followed for at least 3 years for medians and ranges. The last follow-up day was 30 September 2013, and the survival status was confirmed by patients or family contacts. In this study, the duration of overall survival was defined as the time from surgical resection to death or to the date on which the patient was last known to be alive.

### 4.5. Statistical Analysis

All analyses were performed with the statistical package for social science (SPSS) version 18 (SPSS Institute, Chicago, IL, USA). The Pearson’s χ^2^ test was used to test the differences between the cases and the control subjects in the distribution of gender, age, ethnicity, and XRCC4 genotypes. The Spearman *r* test was used to analyze the correlation between XRCC4 genotypes and tumor grade of DIA. As the present study was based on an individually matched design, we did conditional logistic regression to elucidate odds ratios (ORs) and 95% confidence intervals (CIs) for risk of DIA. Kaplan–Meier survival analysis with the log-rank test was used to evaluate the relationship between XRCC4 codon 247 polymorphism and DIA prognosis. Hazard ratios (HRs) and 95%CIs for XRCC4 genotypes were calculated from a multivariate Cox regression model (with step-wise forward selection based on the likelihood ratio test). In this study, a *p*-value of <0.05 was considered statistically significant.

## 5. Conclusions

In summary, to the best of our knowledge, this is the first report investigating an association between XRCC4 codon 247 polymorphism and the risk and prognosis of DIA in Guangxi patients. We have found evidence suggesting that the genotypes of XRCC4 with codon 247 Ser alleles may be correlated with increased risk and poor prognosis for DIA, and that the decreasing NHEJ capacity may play an important role in the tumorigenesis of this type of tumor.

## Figures and Tables

**Table 1.** Demographic characteristics of diffusely infiltrating astrocytoma (DIA) cases and controls.

Characteristics	Controls (*n* = 358)		Cases (*n* = 242)		χ^2^	*p*
	
	*n*	%	*n*	%		
Age (years)					0.317	1.000
≤35	19	5.3	13	5.4		
36–40	23	6.4	16	6.6		
41–45	33	9.2	22	9.1		
46–50	76	21.2	53	21.9		
51–55	102	28.5	68	28.1		
56–60	45	12.6	30	12.4		
61–65	34	9.5	24	9.9		
≥66	26	7.3	15	6.2		
Sex					0.318	0.573
Male	230	64.2	150	62.0		
Female	128	35.8	92	38.0		
Race					0.026	0.872
Han	184	51.4	126	52.1		
Minority	174	48.6	116	47.9		

The mean ± S.D. ages were 50.19 ± 9.36 and 50.25 ± 8.88 for cases and controls, respectively.

**Table 2.** XRCC4 codon 247 polymorphism and DIA risk.

XRCC4		Controls		Cases		OR (95%CI)	*p*
	
		*n*	%	*n*	%		
Genotype	AA b	298	83.2	168	69.4	1	
	AS b	39	10.9	40	16.5	1.82(1.13–2.94) a	1.46 × 10^−3^
	SS b	21	5.9	34	14.0	2.89(1.62–5.15) a	3.22 × 10^−4^
Allele	Ala c	635	88.7	376	77.7	1	
	Ser d	81	11.3	108	22.3	2.25(1.64–3.09)	4.43 × 10^−7^

a Odds ratio (OR) conditional on matched set;

b AA, AS, and SS represent the homozygotes of XRCC4 codon 247 Ala alleles, the heterozygotes of XRCC4 codon 247 Ala and Ser allele, and the homozygotes of XRCC4 codon 247 Ser alleles, respectively;

c Ala represents both heterozygous Ala and homozygous Ala;

d Ser represents both heterozygous Ser and homozygous Ser.

**Table 3.** XRCC4 codon 247 polymorphism and DIA risk stratified by race (Han and Minority), gender (female and male), and age (≤50 years and >50 years).

Variable	Genotype	Control		Case		OR (95%CI) a	*p*
	
		*n*	%	*n*	%		
Race b	XRCC4						
Han	AA	152	82.6	87	69.0	1	
	AS	21	11.4	21	16.7	1.73(0.89–3.34)	0.11
	SS	11	5.8	18	14.2	3.07(1.35–7.00)	7.68 × 10^−3^
	AS/SS	32	17.2	39	30.9	2.16(1.25–3.71)	5.65 × 10^−3^
Zhuang	AA	146	83.9	81	69.6	1	
	AS	18	10.2	19	16.4	1.87(0.93–3.76)	0.08
	SS	11	6.1	16	14.1	2.61(1.16–5.92)	0.02
	AS/SS	28	16.3	35	30.5	2.15(1.22–3.78)	7.89 × 10^−3^
Gender c	XRCC4						
Male	AA	193	83.9	104	69.3	1	
	AS	24	10.4	27	18.0	2.10(1.15–3.84)	1.56 × 10^−2^
	SS	13	5.8	19	12.7	2.81(1.33–5.96)	6.95 × 10^−3^
	AS/SS	37	16.2	46	30.7	2.35(1.43–3.86)	7.82 × 10^−4^
Female	AA	105	82.0	64	69.3	1	
	AS	15	11.5	13	14.1	1.40(0.62–3.15)	0.42
	SS	8	6.2	15	16.5	2.86(1.14–7.19)	2.50 × 10^−2^
	AS/SS	23	17.8	28	30.6	1.92(1.01–3.64)	4.60 × 10^−2^
Age d	XRCC4						
≤50	AA	128	84.8	75	72.1	1	
	AS	17	11.3	18	17.3	1.83(0.89–3.78)	0.10
	SS	6	3.8	11	10.6	3.19(1.13–9.02)	2.85 × 10^−2^
	AS/SS	151	99.9	29	27.9	2.19(1.18–4.06)	1.34 × 10^−2^
>50	AA	170	82.1	93	67.2	1	
	AS	22	10.5	22	15.9	1.86(0.98–3.56)	0.06
	SS	15	7.3	23	16.8	2.76(1.37–5.56)	4.56 × 10^−3^
	AS/SS	37	17.9	45	32.7	2.23(1.35–3.70)	1.88 × 10^−3^

a OR conditional on matched set;

b Likelihood ratio test for interaction of the stratified variable (Han and Minority) and XRCC4 codon 247 genotype was calculated as test for the heterogeneity of ORs across strata (interact term OR = 0.99, *P*_interaction_ = 0.983);

c Likelihood ratio test for interaction of the stratified variable (Male and Female) and XRCC4 codon 247 genotype was calculated as test for the heterogeneity of ORs across strata (interact term OR = 1.17, *P*_interaction_ = 0.709);

d Likelihood ratio test for interaction of the stratified variable (Age: ≤50 years and >50 years) and XRCC4 codon 247 genotype was calculated as test for the heterogeneity of ORs across strata (interact term OR = 1.04, *P*_interaction_ = 0.933).

**Table 4.** XRCC4 codon 247 polymorphism and DIA grade.

	II		III		IV	
			
XRCC4	*n*	%	*n*	%	*n*	%
AA	42	89.4	41	77.4	85	59.7
AS	4	8.5	7	13.2	29	20.4
SS	1	2.1	5	9.4	28	19.9
AS/SS	5	10.6	12	22.6	57	40.3

Spearman *r* test: *r* = 0.261 and *p* = 4.07 × 10^−5^.

**Table 5.** XRCC4 codon 247 polymorphism and overall death risk of DIA.

Variable	HR(95%CI)	*p*
XRCC4		
AA	1	
AS	2.26(1.57–3.24)	9.75 × 10^−6^
SS	5.36(3.51–8.17)	6.42 × 10^−15^
Grade		
II	1	
III	4.27(2.53–7.20)	5.32 × 10^−8^
IV	9.27(5.54–15.51)	2.31 × 10^−17^
